# The Dual Role of Exosomes in Hepatitis A and C Virus Transmission and Viral Immune Activation

**DOI:** 10.3390/v7122967

**Published:** 2015-12-17

**Authors:** Andrea Longatti

**Affiliations:** MedImmune, Granta Park, Cambridge CB21 6GH, UK; longattia@medimmune.com; Tel.: +44-1223-898-224

**Keywords:** exosomes, Hepatitis C virus, Hepatitis A virus, interferon, plasmacytoid dendritic cells, innate immunity, infection, transmission, immune evasion

## Abstract

Exosomes are small nanovesicles of about 100 nm in diameter that act as intercellular messengers because they can shuttle RNA, proteins and lipids between different cells. Many studies have found that exosomes also play various roles in viral pathogenesis. Hepatitis A virus (HAV; a picornavirus) and Hepatitis C virus (HCV; a flavivirus) two single strand plus-sense RNA viruses, in particular, have been found to use exosomes for viral transmission thus evading antibody-mediated immune responses. Paradoxically, both viral exosomes can also be detected by plasmacytoid dendritic cells (pDCs) leading to innate immune activation and type I interferon production. This article will review recent findings regarding these two viruses and outline how exosomes are involved in their transmission and immune sensing.

## 1. Introduction

Exosomes, believed to be nothing more than expelled cellular waste containers after their discovery in the early 1980s [1,2,3], have since been found to act as important intercellular messengers carrying functional RNAs, proteins and lipids that can induce phenotypic changes in recipient cells [4]. Exosomes are created by invagination of the endosomal membrane leading to the formation of multivesicular bodies (MVBs) [5,6]. Many proteins have been found to be involved in exosome/MVB biogenesis and chief among those are the endosomal sorting complexes required for transport (ESCRTs) although ESCRT independent mechanisms for MVB formation have been described [7,8,9]. It is not clear whether different subpopulations of exosomes are created via different mechanisms of MVB biogenesis or whether incorporation of specific exosomal proteins leads to distinct classes of exosome-like vesicles. Machinery known to be involved in viral exosome biogenesis in the context of Hepatitis A and C infection will be discussed below. When an MVB fuses with the plasma membrane its exosomes are released and can travel over short or long distances to eventually bind to target cells and deliver their cargo. What causes an MVB to fuse with the plasma membrane rather than be degraded in the lysosome is not known but certain membrane trafficking machinery such as Rab35 have been shown to be important in exosome release [10]. Exosomes are secreted by almost all cell types and can be found in all bodily fluids but the exact mechanisms of exosomal targeting to specific cell types (if this occurs *in vivo*) and cargo release remain to be elucidated [11,12,13].

## 2. Exosomes Emerge as Important Players in Viral Pathogenesis

Hepatitis C virus (HCV) is a major blood borne human pathogen chronically infecting 130 to 170 million people worldwide [14,15]. HCV infection persists in the majority of exposed individuals and can cause severe liver disease if left untreated. The virus has evolved mechanisms to blunt the innate immune response in infected hepatocytes by cleaving a key intermediate in innate immune signaling via its NS3/4A protease [16,17,18]. None the less, HCV induces type I interferon responses in the infected liver and it has been shown that this occurs via activation of liver-resident plasmacytoid dendritic cells (pDCs), which produce type I interferon when in contact with HCV infected hepatocytes [19]. Further, it has been shown that pDC sensing of infected cells depends on exosomal transfer of HCV RNA from infected hepatocytes to pDCs [20]. This could explain the previously confusing finding that interferon and interferon-stimulated genes (ISGs) are present in HCV infected livers despite the inability of infected cells to produce type-I interferon due to viral escape mechanisms. Recent findings have also shown that HCV induces type-III interferons in infected livers, which leads to expression of the same ISG pattern as type-I interferons and could explain the presence of antiviral responses in infected patients. It is not clear, however, which cell type produces the bulk of the type-III interferons. Although pDCs can produce interferon-III other cells such as type-2 myeloid dendritic cells (mDC2) may be the main source [21,22,23,24]. It is not known whether HCV-exosomes can induce type-III interferons as well as type-I interferons and future work should elucidate this, and, more broadly, the role of type-III interferons in HCV infection.

Subsequent studies could also show evidence of virion-independent exosomal transmission of viral RNA and *de novo* infection of naïve cells [25,26,27,28]. It remains to be elucidated, which process predominates *in vivo*.

**Figure 1:**
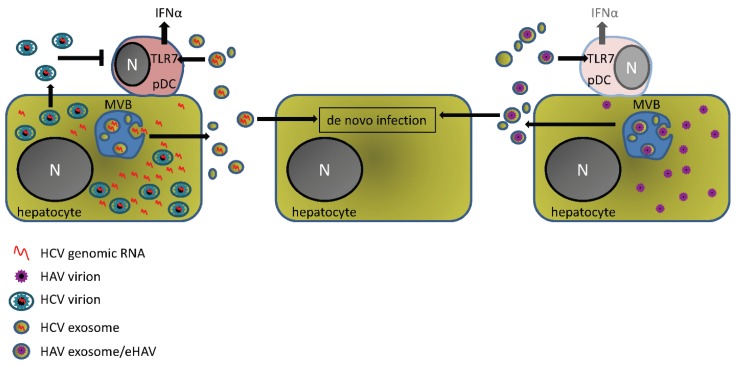
Full-length infectious Hepatitis C virus (HCV) RNA or assembled Hepatitis A virus (HAV) virions can be incorporated into exosomes in infected cells and are secreted into the extracellular milieu. From there viral exosomes can infect other susceptible cells or be internalized by resident pDCs leading to type I interferon production that is dependent on viral RNA recognition by TLR7. Interestingly, while HCV virus particles (or non-enveloped HAV virions) do not activate type-I interferons in pDCs, it has been shown that HVC virions can impair pDC function in infected patients, most likely though viral proteins [29,30,31]. While pDCs are abundant in the HCV infected liver their numbers are much less during HAV infection, which may explain the near absence of type I interferon-stimulated gene expression in HAV infected humans and primates. IFNα, interferon-alpha; TLR7, toll-like receptor 7; N, nucleus; pDC, plasmacytoid dendritic cell; MVB, multivesicular body.

Like HCV, Hepatitis A virus (HAV) is a hepatotropic positive-strand RNA virus that employs similar strategies to inhibit innate immune responses using protease mediated cleavage of innate immune adaptors [32,33,34]. Unlike HCV though, HAV causes moderate to severe acute liver inflammation but is unable to establish persistent infection. HAV is cleared after 3–4 weeks accompanied by the appearance of virus specific antibodies [35,36,37]. HAV has been traditionally described as a non-enveloped RNA virus belonging to the *Picornaviridae* family (as opposed to HCV, which is an enveloped virus of the *Flaviviridae* family) but recent studies have found that the virus particles can be engulfed by host membranes that resemble exosomes and as such are secreted from infected cells in a way that protects the virus from antibody-mediated immune responses [38]. This may explain the previously unsolved finding that both anti-HAV and inactivated virus could prevent disease even when administered after virus replication had been well established [39]. As with HCV, however, these viral exosomes or enveloped HAV particles (eHAV) can also be transmitted to pDCs where a comparable activation of the type I interferon response occurs [40] (Figure 1).

## 3. Exosomal Transport of Infectious Viral Agents and Activation of the Interferon Response

As outlined above, it has been shown that full length HCV RNA can be incorporated into exosomes in infected cells. Although the exact mechanism for RNA incorporation is unknown, this seems to be an active process since HCV RNA is more than a 1000-fold enriched compared to GAPDH mRNA. Since these experiments were done using HCV subgenomic replicon cells that replicate HCV RNA lacking the viral structural proteins core (which forms the viral capsid) and E1/E2 glycoproteins this process does not require full virus assembly [20,41,42]. This indicates that HCV exosomes do not need any viral structural proteins to be transferred to pDCs or infect other hepatocytes. In support of this, it was found that anti-HCV-receptor antibodies that block virus entry and infection such as anti-CD81, anti-SRB1 and anti-ApoE did not inhibit exosomal HCV transmission [26]. What is needed for HCV exosome release, however, are the ESCRT proteins chromatin-modifying protein 4B (CHMP4B) and tumour susceptibility gene 101 (TSG101) as well as the membrane trafficking regulator Annexin A2 (ANXA2), which are all part of the canonical machinery involved in MVB biogenesis and exosome secretion [20]. It is not known how exosomes are taken up by pDCs or how the HCV RNA is released to engage with endosomal TLR7, which is essential for interferon production by the pDCs but close cell-to-cell contact between infected cells and pDCs is essential for efficient innate immune activation. This latter point seems to be a common theme across different viruses although it is not clear how cell-cell contacts between pDCs and infected cells are established in each case. This is reviewed in more detail by Assil *et al.* [43].

In contrast, for HAV it has been shown that exosomally packaged virions termed eHAV and not viral RNA exosomes are responsible for pDC activation and spread of infection. Similar to HCV RNA, HAV virion incorporation into exosomes requires certain components of the ESCRT machinery although they differ slightly between the two viruses. eHAV packaging and release was shown to be dependent on the ESCRT proteins Alix and VPS4B but not on other components like TSG101, HRS, CHMP4A, CHMP4B, and CHMP4C [38,40]. This is particularly interesting since CHMP4B and TSG101 were shown to be essential for exosomal HCV RNA release and further studies should elucidate exact exosome incorporation mechanisms for different viral species. Assuming that HCV RNA and HAV virions are packaged via specific signals and not randomly into exosomes it would be interesting to unravel the different mechanisms responsible for RNA *vs.* virion targeting to MVBs. Interestingly, it has been shown that HCV relies on the ESCRT protein HRS for export of its nucleocapsids, which was dispensable for eHAV release suggesting that while many viruses hijack the ESCRT machinery for their export there are mechanistic differences [44,45].

Both concentrated HCV (which is enveloped) and HAV (which is not enveloped) particles fail to activate pDCs. In both cases, close contact of pDCs with infected hepatocytes or highly concentrated viral exosomes were necessary for induction of interferon-alpha production [20,40]. This suggests that exosomal uptake and cargo delivery, of which we know little, is essential for pDC activation and such mechanisms may play a role in many different viral infections as they are universal to mammalian exosome biology. Activation of pDCs by both types of viral exosomes relies on recognition of the viral RNA by endosomal TLR7 but in either case it is not known how the RNA is released from the exosome (and the HAV capsid) to reach endosomal TLR7. In fact, our knowledge of how exosomes deliver any kind of cargo such as proteins or RNAs to the cytoplasm or to endosomal compartments is incremental at best and further studies are needed to elucidate theses mechanisms, which are important in various diseases as well as viral infections. eHAV is taken up by pDCs via an endocytic mechanism that is facilitated by phosphatidylserine receptors and requires acidification of the endosomes as interferon production was ablated in the presence of chloroquine or bafilomycin A1, which is consistent with our current limited knowledge of non-viral exosome uptake [46,47,48,49,50,51,52]. It is not known how or if HAV RNA is unpackaged from its capsid to reach TLR7 or whether “naked” HAV RNA is co-packaged with HAV virions into exosomes.

It is likely that both HCV and HAV exosomes are taken up by pDCs via an endocytic mechanism involving exosomal surface proteins although such mechanisms have been poorly defined for any kind of exosomes *in vivo* or *in vitro*. It is not clear whether HCV or HAV (non-enveloped) virions are taken up by pDCs via viral or other receptors although it is clear that neither virus can replicate in pDCs nor do they activate interferon. The fact that HCV virus particles can impair pDC function in infected patients suggests that an interaction does occur but why this does not lead to viral RNA uptake and recognition by TLRs in the pDCs is a question that future studies should address.

## 4. Viral Exosomes as Immune Evasive Infectious Particles

Despite the ability of HCV and HAV exosomes to activate innate immune signaling in pDCs both types of vesicles can also infect naïve hepatocytes. In the case of HCV this can be achieved via exosomes containing just the positive-sense viral RNA genome as it can occur in the absence of viral structural proteins [25,27,53]. Although it is difficult to separate HCV viral particles from HCV exosomes due to their similar biophysical properties some experiments suggest that core and E2 proteins can be incorporated into viral exosomes but it is not clear whether this is an active process or happens passively during exosome biogenesis in infected cells and it is certainly not a pre-requisite for exosomal viral transmission. In fact, exosomal transmission of HCV was shown to be independent of canonical viral entry receptors engaged by E1/E2 viral glycoproteins [26]. HCV exosome transmission was also partially resistant to antibody neutralisation using patient derived IgG suggesting that viral exosomes can escape humoral immunity. Interestingly, studies using exosomes purified from HCV infected cells, suggest that mir-122 together with Ago2 and HSP90 are associated with exosomal HCV RNA. This could increase viral exosome infectivity since mir-122, Ago2 and HSP90 have been shown to be important cellular co-factors for HCV replication [26,54,55,56,57,58,59,60,61]. It remains to be seen whether all viral exosomes contain those co-factors or not and whether that may predispose an exosome to be infectious rather than activate a pDC. Finally, Bukong *et al.,* have also found that HCV negative-sense RNA, a replication intermediate, can be found in exosomes of treatment non-responders although the significance of this remains to be elucidated.

HAV exosomes, or eHAV, have also been shown to be highly infectious circulating particles. In fact, eHAVs and not non-enveloped HAVs seem to be the predominant form of circulating viral particles in infected humans and chimpanzees. Unlike HAV, eHAV particles are resistant to neutralizing antibodies, which may explain the late appearance of virus-specific antibodies in infected patients [36]. Non-enveloped virus can be found in feces, which may be due to stripping of the membrane during passage through the biliary tract from the liver to the gut. These findings raise questions about broadly categorizing viruses into enveloped and non-enveloped species and it will be interesting what future research can tell us about hijacking of exosomes by various viruses.

## 5. Discussion

The role of viral exosomes in HCV and HAV infection seems paradoxical since, on the one hand, these exosomes activate the innate immune response via pDCs, and on the other hand they mask infectious viral RNA or particles from antibody-mediated immune responses. It is possible that exosomes evolved as a defense mechanism to activate innate immunity despite viral strategies to block these pathways in infected cells. However, the viruses may have co-evolved to use exosomes to spread undetected by the adaptive immune response. In the case of HAV, the latter certainly seems to be the principal mechanism *in vivo* since eHAV is the predominant form found in the blood of infected patients and chimpanzees and is fully infectious, whereas non-enveloped, classical HAV is found in the feces of infected animals or humans [38]. Furthermore, the disappearance of pDCs from HAV infected livers prior to inflammation suggests that the former is not a major contributor to anti-HAV immune responses during the peak of infection, which is not accompanied by strong type I interferon regulated responses.

HCV infection on the other hand leads to sustained inflammation of the liver characterized by induction of type I and type-III interferons and an abundance of pDCs. Cell culture experiments using subgenomic replicon cells suggest that exosomal HCV infection is inefficient compared to free virus infection [25]. The contribution of exosomal HCV *vs.* free virus infection *in vivo* is difficult to determine since, unlike HAV, the viral particles exhibit a similar size and density to viral exosomes and contamination of exosome preparations with free virus is difficult to exclude. However, using an immune-isolation protocol to try and largely separate virus from viral exosomes Bukong *et al.,* show that HCV-exosomes can be found in the circulation of infected patients and that these vesicles are infectious and largely resistant to neutralization by anti-HCV-E2 antibodies [26,27]. It is possible that these HCV-exosomes constitute an immune-protected viral reservoir that allows the virus to persist after antiviral treatment or liver transplantation whereas the main spread of infection in the liver occurs via free HCV virus.

It is not known how HCV (or eHAV) exosomes are internalized by pDCs but several well described HCV entry receptors have been found to be dispensable for exosomal HCV transmission to hepatocytes. Never the less, exosomal HCV spread (like HCV viral spread) seems to retain exclusive tropism for hepatocytes. There are several possible explanations for these findings that should be addressed in future investigations. Strong post-entry restrictions may prevent viral replication and release in cells other than hepatocytes. Alternatively, the very low infectivity of HCV exosomes compared to HCV virus (at least *in vitro*) may prevent infection of cell types other than hepatocytes, which represent the natural, and therefore likely the optimal host cell type of HCV. Lastly, it is also possible that HCV exosomes only exhibit auto- or paracrine transfer mechanisms although they can be found in the blood stream of infected individuals.

It is becoming increasingly clear that exosomes play an important role in many viral infections [11]. In the case of HCV and HAV infections, we know now that viral exosomes can contribute to both viral immune evasion by masking viral particles or genomes, as well as activation of pDCs and innate immune responses. Full-length HCV RNA without viral proteins or complete HAV viral particles are incorporated into exosomes using the standard cellular exosome machinery and secreted to the extracellular space. From there, the viral exosomes are either taken up by pDCs or susceptible hepatocytes leading to innate immune activation or *de novo* infection, respectively. In the case of HAV infection the latter seems to be more important since pDCs largely disappear from infected livers at the peak of infection, whereas in the case of HCV infection pDC activation characterized by type I interferon production may be a major contributor to liver inflammation and disease progression. Although type I interferons are generally considered to be a major first line of host defense after viral infection recent studies suggest that sustained interferon production may in some way contribute to viral persistence [62,63,64]. HAV and HCV infections seem to support this paradoxical idea since the former is not accompanied by type I interferon responses and never persists in infected individuals whereas the latter becomes chronic in most patients and is characterized by strong induction of type I and type III interferons. It will be interesting to see what mechanistic insight future studies can give us about these observations and how these observations extend to other viruses. For example, similarly to HCV, lymphocytic choriomeningitis virus (LCMV) can persist in the presence of antiviral immune responses characterized by type I interferon induction, which is mediated by pDC uptake of viral exosomes containing LCMV RNA [65]. For other viruses, such as human immunodeficiency virus (HIV), the role of exosomes may be equally complex as evidence suggests that exosomes facilitate both enhancement and inhibition of viral spread depending on the cell type of origin [11]. TLR7 dependent pDC activation by HIV infected lymphocytes has been demonstrated and it will be interesting to investigate the role of exosomes in this process [66]. Taken together, these findings certainly warrant further investigation into the role of viral exosomes in innate immunity and immune evasion.
